# Large-area soft-imprinted nanowire networks as light trapping transparent conductors

**DOI:** 10.1038/srep11414

**Published:** 2015-06-19

**Authors:** Jorik van de Groep, Dhritiman Gupta, Marc A. Verschuuren, Martijn M. Wienk, Rene A. J. Janssen, Albert Polman

**Affiliations:** 1 Center for Nanophotonics, FOM Institute AMOLF, Science Park 104, 1098 XG Amsterdam, The Netherlands; 2 Departments of Applied Physics and Chemical Engineering & Chemistry, Eindhoven University of Technology, 5600 MB, Eindhoven, The Netherlands; 3 Philips Research Laboratories, High-Tech Campus 4, 5656 AE Eindhoven, The Netherlands

## Abstract

Using soft-imprint nanolithography, we demonstrate large-area application of engineered two-dimensional polarization-independent networks of silver nanowires as transparent conducting electrodes. These networks have high optical transmittance, low electrical sheet resistance, and at the same time function as a photonic light-trapping structure enhancing optical absorption in the absorber layer of thin-film solar cells. We study the influence of nanowire width and pitch on the network transmittance and sheet resistance, and demonstrate improved performance compared to ITO. Next, we use P3HT-PCBM organic solar cells as a model system to show the realization of nanowire network based functional devices. Using angle-resolved external quantum efficiency measurements, we demonstrate engineered light trapping by coupling to guided modes in the thin absorber layer of the solar cell. Concurrent to the direct observation of controlled light trapping we observe a reduction in photocurrent as a result of increased reflection and parasitic absorption losses; such losses can be minimized by re-optimization of the NW network geometry. Together, these results demonstrate how engineered 2D NW networks can serve as multifunctional structures that unify the functions of a transparent conductor and a light trapping structure. These results are generic and can be applied to any type of optoelectronic device.

High-quality transparent conducting electrodes (TCEs) form an essential component of a broad range of optoelectronics devices including LEDs, displays, and solar cells. For solar cells, the inclusion of a transparent conductor is particularly important when the charge carrier diffusion length is short, such as in for example Si heterojunction, perovskite or organic cells. The most commonly used TCE is indium-tin-oxide (ITO). However, high material costs^1^,^2^, the scarcity of indium^3^, brittleness^4^,^5^, optical absorption^6^,^7^ and incompatibility of the sputtering process with organic layers^8^ strongly motivate the development of a replacement for ITO.

The high conductivity of metals has stimulated interest in metal nanowire (NW) networks and meshes as alternatives to ITO. A wide variety of geometries have been proposed, including random nanowire meshes^7^,^9^,^10^,^11^,^12^, percolated films^13^,^14^, 1D (nano) imprinted gratings^4^,^5^,^15^, nanogratings interconnected with mesoscale wires^16^, self-assembled microstructures^17^, as well as NW-graphene hybrid structures^18^. These nanoscale and multiscale geometries can be designed to provide improved optoelectronic performance relative to ITO, achieving concurrent improvements in both optical transparency and electrical conductivity. Furthermore different metals can be used, which provides tuneability of the workfunction of the contact, and allows for inverted fabrication schemes.

Plasmonic light trapping effects can further improve the absorption in thin absorber layers. For organic photovoltaic devices, plasmonic light trapping has recently become the subject of intense interest due to the short carrier diffusion lengths in these material systems. To facilitate efficient carrier extraction the active layer thickness must be thin, however this limits the optical path length inside the absorbing material. Optical efficiency enhancements have already been demonstrated^19^ by employing both localized plasmon resonances^20^ and surface plasmon polaritons (SPPs) on the (rear) electrode^21^,^22^,^23^. ITO can be replaced with a conductive plasmonic array made of 1D silver gratings^4^,^24^ and silver nanohole arrays^25^. Random NW networks provide limited light trapping capabilities through random scattering^26^. However, all these geometries are either strongly polarization dependent (limited or no light trapping for other polarization) or allow no control over the network geometry.

Recently, we have shown using e-beam lithography (EBL) that 2D networks of silver NWs can match the optical performance of ITO as a transparent conductor, while offering significantly improved sheet resistances^27^. Unlike random networks, controlled network geometries allow engineered spectral transmission by optimizing the effects of excitation of localized and propagating surface plasmon modes, scattering and coupling to guided modes in an underlying semiconductor substrate.

In this work, we employ soft-imprint lithography^28^ to transfer this small-area concept into large-area applications of NW networks. The facile fabrication of large-area NW networks allows us to systematically vary NW width and pitch and study the influence on spectral transmittance and sheet resistance, and to demonstrate centimeter-scale NW network based functional devices. Furthermore, we employ the engineered 2D NW networks to systematically study plasmonic light trapping in an organic solar cell in a fully controlled manner. We demonstrate the unique combination of both mode-matched light trapping and charge collection in a single multifunctional layer using P3HT-PCBM polymer solar cells. The results from this well characterized model system^19^,^29^ are generic, and applicable to all thin film devices.

## Results And Discussion

### Nanoimprinted nanowire networks as transparent conducting electrodes

Substrate conformal imprint lithography (SCIL) is a high-resolution nanoimprint technique that employs a bilayer PDMS stamp to reproducibly transfer high-resolution nanopatterns onto substrates in a fast, facile and inexpensive manner^28^. Here, we use this technique to fabricate Ag NW networks over centimeter-scale areas on a glass substrate with nanometer control over nanowire position, dimension, and spacing (Supplementary Fig. S1). Briefly, a PMMA sacrificial layer and a silica sol-gel layer are deposited on a glass substrate by spin coating. A 6” diameter SCIL stamp containing the nanowire pattern is applied and, after 30 minutes of drying in ambient conditions, removed to leave behind the patterned silica sol-gel layer. Subsequent reactive ion etching of the PMMA, thermal evaporation of Ag through the sol-gel/PMMA apertures, and a liftoff process complete the fabrication of networks of Ag NWs. While this process can be applied to wafer-scale processing, here the stamped pattern consists of 40 square networks (2 × 2 mm^2^ each), with each square containing a 30 nm high network with different NW width and pitch (widths: 55–130 nm, pitches: 300–1000 nm in steps of 100 nm). Electrical contact pads on either side of each network (125 μm × 2 mm) were fabricated using UV-lithography, followed by thermal evaporation (5 nm Cr, 50 nm Au), and subsequent lift-off (see sketch in Supplementary Fig. S2a).

**Figure 1:**
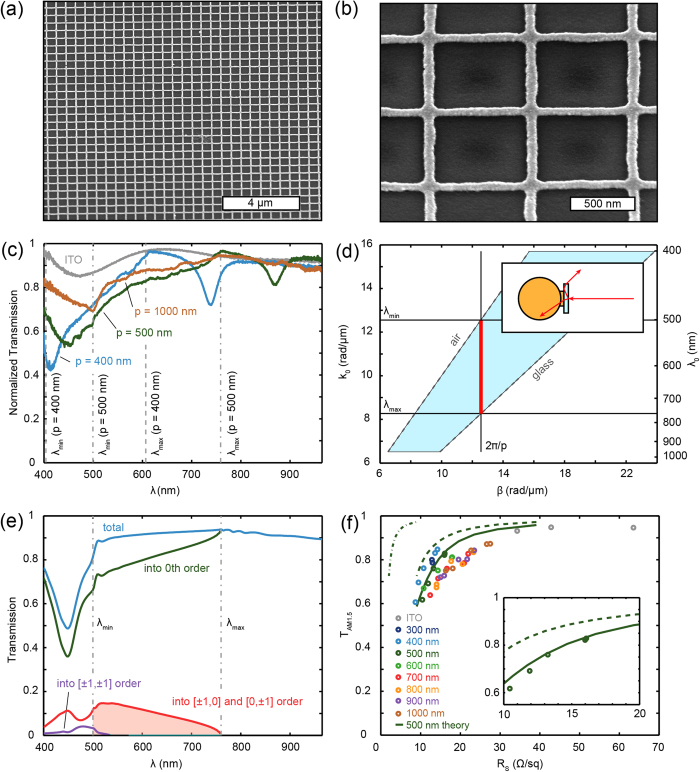
Nanoimprinted silver nanowire networks as transparent electrodes. (**a**) SEM image of large-area 500 nm pitch Ag NW network on glass. (**b**) High resolution SEM image of 800 nm pitched network with NW width ~85 nm. (**c**) Measured normalized transmission of thinnest NWs with 400 (blue), 500 (green) and 1000 nm (orange) pitch (~60, 65 and 80 nm width respectively). Also shown is the transmittance of a 200 nm layer of ITO (gray). Vertical lines indicate the spectral range where mode coupling occurs for *p* = 400 nm (dashed) and *p* = 500 nm (dash-dot). (**d**) Dispersion diagram for 500 nm pitched network, showing light lines in air and glass. The wavelengths for which coupling to guided modes occurs are indicated by the red line. Inset: sketch of integrating sphere setup. (**e**) Simulated transmittance into different diffraction orders for NW network with 65 nm width, 30 nm height and 500 nm pitch. The shaded area corresponds to the power coupled to guided modes (average 5%). (**f**) Average transmission (weighted for AM1.5 photon flux) versus sheet resistance of all fabricated NW networks. The different pitches are indicated with different colors. The measured data for 200, 150 and 110 nm thick layers of ITO (light gray, from left to right) are also shown. The green lines show theoretical data for *p* = 500 nm: total transmission and theoretical resistivity (dash-dot), total transmission and actual resistivity (dashed), transmission excluding light coupling to guided modes and actual resistivity (solid). The inset shows the match to the experimental data for *p* = 500 nm.

The NW networks resulting from this top-down SCIL process are uniform over large areas (Fig. 1a), with the wires exhibiting both smooth interfaces and high-quality interconnections (Fig. 1b). Unlike chemically synthesized random NW network meshes, these large-area NW networks are fabricated out of a single metallic sheet with correspondingly low inter-wire junction resistance, as we will show. We use 40 different combinations of pitch and NW width, each with a different metal filling fraction, to explore the trade-off between high optical transmittance and low sheet resistance on each array geometry.

The performance of these NW networks as transparent conducting electrodes was first characterized by measuring both the white-light transmittance and the lateral electrical sheet resistance. Optical transmission spectra were taken of the Ag NW coated glass, with the NWs at the front (incident) side. An integrating sphere was used to allow the collection of light diffracted out of the sample under large angles (inset Fig. 1d). To isolate the influence of the NW networks, the measured transmission spectra were normalized to the transmittance of a bare glass sample.

All nanowire network spectra exhibit broadband normalized transmittance as a result of guided modes through the apertures^2^,^27^, with two main perturbations (Fig. 1c). The sharp dip in the red spectral range is a result of diffractive coupling of light to propagating surface plasmon polaritons (SPPs) along the nanowires^27^. A broad dip in the blue spectral range originating from absorption due to the excitation of the transverse local surface plasmon resonance (LSPR) of the individual wires; and plasmonic light scattering into waveguide modes in the glass substrate.

This diffractive coupling of light into the glass substrate, appearing as a reduction in the transmission spectra (Fig. 1c), is a desired feature in light trapping geometries. In a functional device, the guided light couples to the absorber layer and will enhance the absorption, as demonstrated below. To investigate it further we plot the maximum in-plane wavevector 
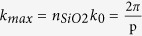
 that can be obtained in a glass substrate through scattering off a grating with pitch *p* (Fig. 1d, where *n*_*sio2*_ = 1.52). Guided modes lie between the light lines in air and in the glass substrate (Fig. 1d, shaded region). Combined, these conditions define the wavelength range for which mode coupling occurs (red line in Fig. 1d). For optical frequencies where *λ*_*min*_ < *λ*_0_ < *λ*_*max*_ (dashed vertical gray lines in Fig. 1c) light couples to guided modes in the glass substrate; the density of these optical modes decreases with increasing wavelength, resulting in reduced coupling and a concomitant increase in transmittance through the slide, as is observed.

Finite-difference time-domain (FDTD) simulations^30^ were performed to calculate the fraction of light trapped by mode coupling into the glass substrate (Supplementary S3). To this end, we simulated the transmittance of the top interface with Ag NW network (neglecting the 1 nm Ge seed layer, see Supplementary S6), and calculated the angular distribution of the light transmitted into the substrate. For the experimental Ag network dimensions (*w* = 65 nm, *p* = 500 nm), the results are shown in Fig. 1e. The spectral distribution of total transmittance into the substrate (blue) is separated into the 0^th^ order transmittance (green), and the [ ±1,0] and [0, ±1] (red) and [ ±1, ±1] (purple) diffraction orders in the substrate. Since the simulations only consider the top interface, direct comparison with the measured spectrum (Fig. 1c) in absolute terms is incorrect. However, comparing the 0^th^ order transmittance (Fig. 1e, green line) with the measured spectrum (Fig. 1c, green line) shows good qualitative agreement. The dip in transmission as a result of plasmon absorption (*λ*_0_ ~ 450 nm), the gradual increase in transmittance due to substrate modes (500 ≤ *λ*_0_ ≤ 760 nm), as well as the kink in transmission at λ_0_ = 760 nm are clearly reproduced.

From the angular analysis we find that 5% of the photons in the AM1.5 solar spectrum will couple to guided modes, indicated by the shaded region. In the experiment, this fraction is not collected giving rise to the kink in the measured transmittance at λ_*max*_ = 760 nm (see Fig. 1c,e). Figure 1e shows that the total transmitted spectrum (0^th^ order plus grating coupling into the substrate) is ~ 90% over the entire spectral range from 500–900 nm (88% averaged over photon flux). This is very similar to the average transmittance through the ITO layers for this spectral range (Fig. 1c) despite the large area fraction covered by metal (20–31%, as determined from SEM images). Note that the simulated reflection and parasitic losses in the metal are limited to only 9.7% and 2.3% respectively (averaged over photon flux).

The sheet resistance (*R*_*s*_) of the NW networks was characterized using four-point-probe measurements. Two probes were placed on each contact pad, and the resulting current-voltage (IV) response measured (see Supplementary Fig. S2). For the ITO, the standard geometry of four equally spaced probes was used to measure *R*_*s*_. All NW networks and ITO samples showed Ohmic behavior, allowing *R*_*s*_ to be extracted from a linear fit.

For all nanowire networks the sheet resistance was measured and found to be significantly lower than the resistance of the ITO reference samples (thicknesses 110, 150 and 200 nm). The data is plotted in Fig. 1f for different pitches (shown as different colors) with the corresponding measured average transmittance (*T*_*AM*1.5_), weighted for photon density in the AM1.5 solar spectrum (using the experimental spectral range; 400 < λ < 965 nm). For comparison, the measured data for ITO is shown in gray. The NW networks had a measured *T*_*AM*1.5_ from 40–87% compared to 93–95% for commercially available ITO. Note that this neglects the  ~ 5% coupled by the NW networks into waveguided modes of the glass substrate. However, the NW networks show sheet resistances in the range of 8.7–27.5 Ω/sq, which is much lower than ITO studied here (34.3–63.6 Ω/sq). This shows that, despite the nanoscale dimensions of the wires, the presence of the highly conductive Ag metal permits a large improvement in sheet resistance.

Comparing the measured data with the theoretical sheet resistance for a square grid we find that the effective resistivity of the material is (7.0 ± 0.5) × 10^−8^ Ωm, which is 4.3 ± 0.3 times larger than the bulk resistivity of silver. We attribute this to variations in wire width, surface roughness, and electron scattering from grain boundaries. This is a significant improvement compared to the 5.7 times increase in resistivity observed for NW networks fabricated by e-beam lithography^27^, which we attribute to reduced surface roughness on the individual NWs.

To determine the upper limit in performance that can be achieved with the NW networks, as well as to correct for the fraction of light coupled to guided substrate modes, we simulated the transmittance for *p* = 500 nm, *h* = 30 nm and *w* = 30–130 nm (similar to Fig. 1e), converted the spectra to normalized transmittance (Supplementary S3), and calculated the sheet resistance. First, the theoretical absolute maximum performance for the *p* = 500 nm networks (Fig. 1f, dash-dotted line) is obtained from the total transmission and sheet resistance calculated from the bulk resistivity of Ag. Second, the performance of the NW networks including the light coupled to guided modes (dashed line) is calculated by using the realistic resistivity (4.3 times bulk resistivity) instead of bulk resistivity. Third, by excluding the light coupled to guided modes the experimental conditions used in this work are calculated (solid line). Indeed, the theory line (solid) shows good agreement with the measured results (see inset of Fig. 1f), showing that the simulations accurately describe the experiments. Finally, we can compare the performance of the NW networks (using full transmission, dashed line) with that of ITO. We find that the NW networks outperform the transmittance and sheet resistance of ITO.

To summarize the performance as a transparent conductor, we calculated the figure of merit (FoM) for transparent conductors, defined as the ratio of electrical conductivity to optical conductivity^9^,^31^,^32^


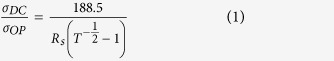


The FoM for the *p* = 500 nm NW network is calculated using the theory line for full transmission (Fig. 1f dashed, FoM = 120–335) as well as excluding the light coupled to guided modes in the substrate (Fig. 1f solid, FoM = 68–223). Both show a higher FoM than that of ITO (105–162).

Figure 1f clearly shows the tradeoff between high transmittance (thin wires, large pitch) and low sheet resistance (wide wires, small pitch). Tuning these parameters – pitch and width – allows the selection of a desired resistance/transmittance combination that can be optimized for specific applications as well as tuning the spectral range of plasmonic enhancement. Note that the spectral range of the plasmonic response strongly depends on the dielectric surrounding, as will be shown below.

### Nanowire network based polymer solar cells

Next, we demonstrate the application of these NW networks as the TCE in functional organic solar cells. Starting with the NW networks printed on glass, P3HT-PCBM polymer solar cells were fabricated on the networks in a superstrate configuration. This well-established cell type was chosen to serve as a model system where ITO serves as the transparent conductor^29^. Also, with the absorption edges of P3HT around *λ* ~ 650 nm^29^,^33^ and that of PCBM at *λ* ~ 700 nm, this type of cell can benefit significantly from light trapping in the red and infrared spectral regions where both direct absorption and charge-transfer (CT) absorption are weak.

**Figure 2:**
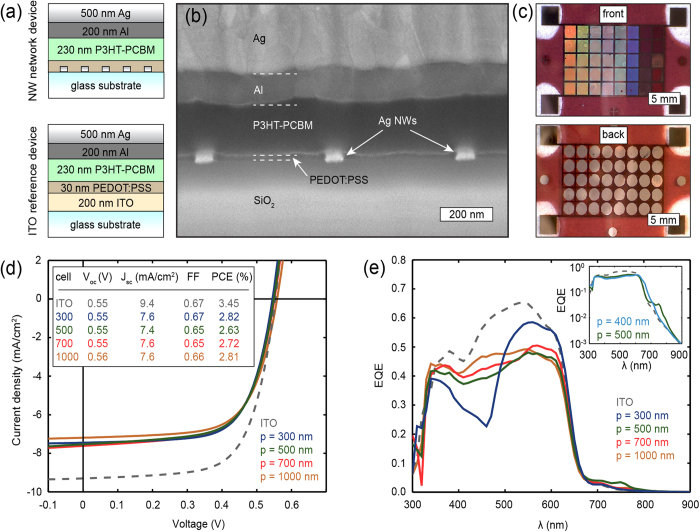
Silver NW network based polymer solar cells. (**a**) Schematic cross sections of the layer stack of the NW network device (top) and ITO reference device (bottom). (**b**) FIB cross section of a device with *p* = 500 nm, clearly showing the Ag NW network embedded in the cell. (**c**) Photograph of front (top) and back (bottom) of the completed devices, showing the 40 different cells on one substrate. (**d**) Measured IV-curves for a 300 (blue), 500 (green), 700 (red) and 1000 nm pitch device (orange). Silver wire widths were ~65 – 85 nm. Also shown is the data for the ITO reference device (gray, dashed). The cell parameters are shown as an inset. The *J*_*sc*_ listed here is obtained from the EQE measurements. (**e**) Measured EQE for the same devices. The inset shows the EQE for *p* = 400 nm (blue) and *p* = 500 nm (green), and for ITO as a reference (gray, dashed), to emphasize the EQE enhancements in the weakly absorbing spectral range.

The experimental cell geometry is shown in Fig. 2a. Substrates with NW networks were first coated with a 30 nm layer of PEDOT:PSS as a hole-conduction layer. Next, 230 nm of P3HT-PCBM (1:1 weight ratio) was spin coated, followed by thermal evaporation of 1 nm of LiF to modify the work function of the electrode and facilitate electron collection, 200 nm Al and 500 nm of Ag. The PEDOT:PSS and active polymer layer thicknesses were optimized using transfer matrix calculations to maximize optical absorption in the active layer of a flat reference cell (note that this is not the optimum for the NW network devices). The standard TCE reference device used a 200 nm thick layer of ITO (*T*_*AM*1.5_ = 93.1%, *R*_*S*_ = 34.3Ω/*sq*) instead of the Ag NW networks.

Contacting the nanowire networks was achieved using evaporated gold contact pads patterned by UV lithography. The geometry of the front contact is clearly visible through the glass substrate (Fig. 2c, front), where the color of individual squares is the result of scattering from the NW networks. Electrical contacts are made by positioning a probe on one of the 4 corner pads (black squares). The rear contacts (LiF, Al and Ag) are evaporated through a circular physical mask (1.9 mm diameter) such that isolated cells are defined with an area of 0.0284 cm^2^ each (Fig. 2c, back). The layered structure of these cells can be seen in Fig. 2b, which shows a focused-ion beam (FIB) cross section of a representative device. The figure clearly shows the layers including the NWs. Importantly, the 30 nm thin PEDOT:PSS layer conformally coats the NWs, which we found was essential to prevent voltage reduction from current leakage. In total, 3 samples, containing 113 complete cells were fabricated, with an overall yield of 89%. Device failures were a result of current leakage induced by metal residues from incomplete lift-off processes.

To characterize the performance of the Ag nanowire contacted solar cells we performed current density - voltage (*JV*) measurements under white light illumination (100 mW/cm^2^, 1 sun). Typical results are shown in Fig. 2d for NW network-based cells with wire widths of ~ 65–85 nm and pitches ranging from 300–1000 nm (colored lines), along with reference results for an ITO-based cell (gray dashed line). Performance parameters for these cells - open-circuit voltage (*V*_oc_), short-circuit current density (*J*_sc_, obtained from EQE measurements below), fill factor (FF), and power conversion efficiency (PCE) - are shown as an inset.

These *JV* measurements prove that the NW networks function as efficient transparent conductors. The application of the NW networks as a transparent conductor induces no current leakage, as *V*_oc_ is ~ 550 mV for all NW network cells, equal to that of the ITO reference cell. Also, the fill factor, which represents the electrical quality of the device, is ~ 0.66 for all NW network devices, similar to that of the ITO reference device. So, despite the corrugation of the cell surface resulting from conformal coating of the NW network, the networks do not induce additional carrier recombination. Replacing the ITO with NW networks causes a reduction in the short-circuit current density (*J*_sc_) from 9.4 mA/cm^2^ to 7.4–7.7 mA/cm^2^ for the NWs relative to ITO. This difference in *J*_*sc*_ can be attributed to several effects. First, the NW networks used here have slightly lower transmittance than the ITO reference samples (Fig. 1f). Despite the lower sheet resistance, this results into reduced current generation. Second, the active layer thickness is not optimized for the NW network cells. Third, in the solar cell geometry the NW networks are coated by PEDOT:PSS instead of air. As a result, the refractive index contrast at the position of the NWs is reduced from 1 (air) → 1.52 (glass) to 1.52 (glass) → 1.7 (PEDOT:PSS). This reduces the forward scattering of the NWs and thereby increases the reflection of the cell, as confirmed by reflection measurements on the completed devices (see Fig. S4). Fourth, the higher refractive index red-shifts the LSPR to longer wavelengths where the solar flux is higher, thereby increasing the absorption in the NWs. From FDTD simulations we estimate the combined absorption losses (400 ≤ *λ* ≤ 650 nm) in the PEDOT:PSS and nanowire network to be 17.4% and 10.7% with and without the 1 nm Ge layer present, respectively (see Supplemental S6 for influence of Ge). The difference in transmittance can be readily minimized by optimizing the nanoimprint process for thinner wires, as shown in Fig. 1f. The effects of the increased refractive index however, are inherent to the device structure.

To characterize the spectral dependence of cell performance, the external quantum efficiency (EQE) was measured by illuminating the cells with a 1 mm diameter spot of monochromatic light (300 – 900 nm, 10 nm steps) and measuring both the illumination power and resulting photocurrent. The band edge for absorption in the P3HT donor molecule is observed at λ ~ 650 nm (Fig. 2e), in agreement with literature values^29^,^33^. In the blue spectral range the NW network cells show a reduced EQE relative to the ITO cell, due reflection (see Fig. S4b) and absorption in the metal. Furthermore, a clear transition in the shape of the EQE curves can be observed with increasing pitch. For the 300 nm device, the EQE is strongly asymmetric, showing low EQE in the blue and high EQE in the red spectral range. This is a clear sign of strong scattering by the LSPR of the NW network around λ ~ 450 nm. Fano-interference with the non-scattered light causes destructive interference for *λ* *<* λ_*LSPR*_, and constructive interference for λ > λ_*LSPR*_.^34^ For larger NW pitches, the wire density decreases, such that the scattering amplitude of the LSPR also decreases. This reduces the spectral asymmetry, and results in a more spectrally flat response.

Finally, small but significant peaks can be observed in the EQE of the NW devices for λ > 650 nm, exceeding the EQE of the ITO reference device. To show this more clearly, a logarithmic plot is shown in Fig. 2e (inset) for a 500 nm (green) and 400 nm (blue) pitch device. An EQE enhancement is observed for 700 < *λ* < 900 nm and 650 < *λ* < 800 nm for these two devices, respectively. We attribute this EQE enhancement to light trapping as a result of the NW networks scattering light into the guided modes of the polymer cells. This EQE enhancement occurs in the weakly absorbing spectral range of the polymer, corresponding to PCBM (acceptor molecule) absorption up to *λ* *~* 750 nm, and CT absorption up to *λ* ~ 1100 nm^35^.

### Light trapping through NW network scattering

**Figure 3:**
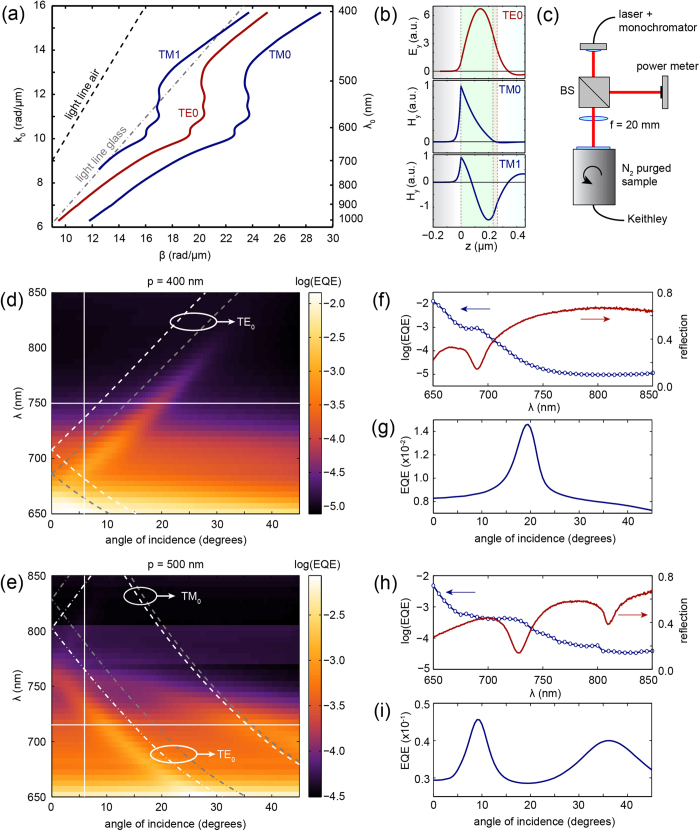
Light trapping in P3HT-PCBM by coupling to guided modes. (**a**) Dispersion curves of the guided modes (TE in red, TM in blue) supported by the layer structure shown in Fig. 2a (top). Also shown are the light lines in air and glass (gray dashed lines). (**b**) Corresponding field profiles of the in-plane electric (for TE mode) and in-plane magnetic (for TM modes) components. The layer structure is shown in the background (colors). (**c**) Experimental setup used for angle-resolved EQE measurements. (**d**) Measured EQE (color, log scale) for *p* = 400 nm. The dashed lines show the calculated dispersion curves for 220 (white) and 285 (gray) nm thick polymer layers. The solid white lines indicate the cross cuts shown in f and g. (**e**) Measured EQE for *p* = 500 nm. Dashed lines correspond to dispersion curves for 225 (white) and 275 (gray) nm thick polymer layers. (**f**) Cross cut through d) for *θ* = 6 degrees (blue, left axis), and measured reflection spectrum (red, right axis) for *p* = 400 nm. (**g**) Cross cut through d) for *λ* = 750 nm. (h) Cross cut through e) for *θ* = 6 degrees (blue, left axis), and measured reflection spectrum (red, right axis) for *p* = 500 nm. (i) Cross cut through e) for *λ* = 715 nm.

To confirm that the EQE enhancement observed at long wavelengths is a result of mode coupling we first calculate the guided modes supported by the solar cell layer structure. Our calculations were performed using a mode-solver to find the complex wavevectors of the eigenmodes propagating in the plane parallel to the polymer layer^36^. The calculations use the layer stack shown in Fig. 2a (top), assuming semi-infinite Al and glass layers, and neglecting the NWs. Complex dielectric constants were used for all materials, taken from the literature for P3HT-PCBM^37^, PEDOT:PSS^37^, Al^38^, and from spectroscopic ellipsometry measurements for ITO. We find three waveguide modes, of which dispersion curves showing the propagating wavevector *β* (real part of complex eigenmode wavevector) for each free-space wavevector *k*_0_ are shown in Fig. 3a. Also shown are the light lines in air and glass (gray lines). Two of the modes are strongly bound (TM_0_ and TE_0_); their dispersion curves lie below the light lines of air and glass. The corresponding modal field profiles are shown in Fig. 3b (in-plane E-component for the TE mode, in-plane H-component for the TM modes). The TM modes show high field intensity on the Al-polymer interface as a result of surface plasmon polaritons. Light scattering from the Ag NWs can result into efficient coupling to these waveguide modes when the in-plane momentum is matched to that of the guided modes:


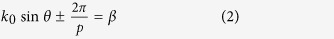


Angle-resolved EQE measurements were performed using the experimental setup is shown in Fig. 3c. Monochromatic radiation is sent through a beam splitter. One beam is weakly focused onto the sample, the other beam is used to measure the illumination power. For each wavelength, a rotation stage is used to scan the angle of incidence from 0 – 45 degrees and the zero-bias photocurrent is recorded.

Figure 3d,e show angle-resolved EQE measurements for pitches of 400 and 500 nm respectively. They show an overall decrease in EQE at wavelengths beyond the absorption edge of the polymer. Both figures show clear bands of enhanced EQE, for which the spectral position shifts with angle of incidence. For normal incidence these EQE peaks occur at ~ 660 nm and ~ 760 nm for *p* = 400 and 500 nm pitches, respectively, in agreement with the inset in Fig. 2e. For *p* = 500 nm, a second branch can be observed for 700 < λ < 750 nm at larger angles of incidence. This dispersive, angle-dependent behavior directly proves coupling of light scattered by the Ag NW networks to waveguide modes in the polymer layer, in agreement with equation (2).

Figure 3e also shows that coupling to the TE_0_ mode is more pronounced than to the TM_0_ mode. This can be explained by the anisotropic refractive index which influences the efficiency of light trapping. The electric field polarization must be aligned with the strongly absorbing axis in order to maximize light trapping efficiency causing that TE modes absorb more efficiently than either the TM and plasmon modes^39^. However, such a difference in mode amplitudes may also be influenced by strong absorption in the Al (Fig. 3b, TM_0_ mode), as well as differences in coupling efficiency.

These experimental results are in good qualitative agreement with calculated dispersion curves for the TM_0_ and TE_0_ modes (Figs. 3d,e, dashed lines). However, an offset is observed for all lines, which can be attributed to three factors. First, the calculations assume perfectly flat layers, while the fabricated devices show variations in thickness (Fig. S3b). Using FIB cross sections we measured the range of polymer layer thicknesses, and observed thickness variations on the order of 65 nm. Dispersion curves calculated for both the minimum and maximum layer thicknesses are shown in Fig. 3d,e. Second, the calculations neglect the presence of the NWs. Third, the calculations assume a dispersive but isotropic refractive index for P3HT-PCBM; the actual dielectric is known to be anisotropic as a result of vertical segregation^33^.

The EQE response of the cells can be further understood by comparing crosscut spectra (taken from Fig. 3d,e) with measured reflection spectra. The total reflection of the completed devices was measured using an integrating sphere setup (see Supplementary S5). The angle of incidence is set to 6 ± 1 degrees to prevent the specular reflection from escaping the sphere. A broadband high reflection can be observed above the band edge (Fig. 3f,h, right axes) for both *p* = 400 nm and *p* = 500 nm due to the low absorption of the polymer. However, clear dips in reflection can be observed around *λ* = 690 nm for *p* = 400 nm and around *λ* = 730 nm and *λ* *=* 810 nm for *p* = 500 nm device.

Comparing the reflection spectra with the measured EQE confirms that the absorption in the active polymer is enhanced by coupling to guided modes. First, crosscuts along wavelength axis show a clear peak in EQE where equation (2) is satisfied, corresponding to an ~1.7 and ~1.5 enhancement for devices with *p* = 400 and 500 nm pitches, respectively (Fig. 3g,i). Figure 3i clearly shows coupling to both the TE_0_ and TM_0_ modes at different angles of incidence. Second, crosscuts along angle axis (*θ* = 6 degrees) show small but clear shoulders on the rapidly decaying background signal above the absorption edge (Figs. 3f,h, left axes). Third, the spectral position of the enhanced EQE shows good agreement with reflection minima, demonstrating a concomitant increase in absorption. Note that the two dips observed in Fig. 3h are in fact due to the same mode propagating in opposite directions, spectrally separated due to the non-zero angle of incidence.

Finally, the field profile of the guided mode was obtained from full wave FDTD simulations^30^. The simulation (using *p* = 500 nm) shows clear coupling to a guided mode at *λ* = 785 nm, with the maximum field intensity located in the polymer layer (see Supplementary S3 and S4 for simulation details and field profile respectively). The spectral position of the guided mode shows good agreement with the measured EQE at normal incidence (Fig. 3e), and both the propagation vector and the mode profile of the guided mode confirm that light couples to the TE_0_ mode.

These results demonstrate how engineered 2D Ag NW networks can serve as multifunctional structures that unify the functions of a TCE and a light trapping structure. Concurrent to the observation of direct evidence for engineered light trapping, the NW networks geometries used in this work resulted into an overall decrease in photocurrent, and thereby power conversion efficiency. The net increase in photocurrent as a result of light scattering and trapping is counteracted by a larger increase in reflection and absorption losses. These losses can be minimized by further reducing the NW width. As shown in Fig. 1f, reducing the NW width from the 65 nm used in this work to 30 – 40 nm will significantly increase the transmission. By adjusting the physical parameters of the networks, these Ag NW TCEs can be optimized for efficient light incoupling and trapping. Such systematic optimization is not possible for random meshes. Recent work demonstrating such optimization has shown significant absorption enhancements in ultra-thin GaAs layers^40^, as well as improved optical transmission into photodiodes using 2D fractal patterns^41^.

## Conclusion

In conclusion, we used nano-imprint lithography for the large-area fabrication of two-dimensional networks of Ag nanowires. The SCIL method provides nanometer control over nanowire position, width, height and pitch. The sheet resistances is as low as 8.7 Ohm/sq for the best conducting network, an average transmission up to 87% is found for the best transmitting networks. Our analysis shows that optimized NW networks outperform the sheet resistance and transmittance of ITO. Using P3HT-PCBM polymer solar cells as a model system, we demonstrated the use of our NW networks as functioning transparent electrodes. The NW network devices showed no losses in *V*_*oc*_ and fill factor, demonstrating good TCE performance. Angle-resolved EQE measurements demonstrate that, in addition to functioning as electrical conductors, the NW networks scatter light into guided modes in the solar cell and enhance absorption. EQE enhancements up to 1.7 are shown in the weakly absorbing spectral range, including the charge-transfer absorption range. Concurrent to the direct observation of controlled light trapping we observe a reduction in photocurrent as a result of increased reflection and parasitic absorption losses; such losses can be minimized by re-optimization of the NW network geometry. This work demonstrates how engineered 2D networks of silver nanowires can simultaneously function as a transparent electrode to replace ITO and as a light trapping layer to enhance the optical absorption.

## Methods

### NW network fabrication

Large glass substrates (69 × 69 x 1 mm) were cleaned with base piranha, followed by a 10-15 min bake-out at 150 °C. Next, ~250 nm PMMA 35 k (300) was spincoated at 1000 rpm in 45 s, followed by a 15 min bake at 150 °C. To make the surface of the PMMA hydrophilic, a 10 s O_2_ descum reactive-ion etch (RIE) was applied. Then, liquid silica sol-gel (home-made) was spin coated at 1000 rpm in 10 s to form a uniform layer of 60 – 70 nm thickness, after which the nano-imprint stamp was applied. After 30 minutes of curing in ambient conditions, the stamp was removed and the nanopatterned sol-gel formed. Next, RIE etching using CHF_3_ (25 sccm) and Ar (5 sccm) was used to anisotropically etch through the residual sol-gel layer (1:45 min, 67 W, 15 mTorr), followed by 12 min of O_2_ descum to etch trough the PMMA and create an undercut.

Thermal evaporation was used to deposit 1 ± 0.5 nm of Ge (0.1 Å/s) and 30 ± 2 nm of Ag (0.5 Å/s). The Ge functions as a seed layer to prevent the Ag from growing in large grains, such that very smooth Ag NWs can be grown^42^,^43^. To perform lift-off, the samples were soaked in acetone at 50 °C for 3 – 5 hours, followed by 10 minutes of megasonication and 1 min of ultrasonication. Finally, the samples were rinsed in isopropanol and dried using nitrogen.

The samples were then coated with S1813 (2000 rpm, 32 s) and baked at 110 °C for 2 min to protect the NW networks during the cutting of the samples to a smaller size (30 × 30 mm), in order to be compatible with the rest of the processing. After the cutting, the samples were cleaned by rinsing in acetone and isopropanol, followed by blow-drying.

UV-lithography was used to fabricate the Au contacts for the NW networks. After a 5 min bake-out at 100 °C, an HMDS primer was applied (4000 rpm, 32 s) for adhesion, followed by a 1 min bake at 100 °C. A negative-tone resist (ma-N 1410, 1400 rpm, 32 s, ~925 nm thick) was spincoated, followed by a 90 s bake at 100 °C. The samples were then exposed through a flexible foil mask (Selba) for 90 s at 5 mW (450 mJ dose) to create 125 μm × 2 mm large contacts that have 50 μm overlap with the NW networks. To develop, the samples were dipped in ma-D 533S developer for 85s, dipped in water twice to stop the development and dried using nitrogen. Again, thermal evaporation was used to deposit 5 ± 1 nm of Cr as an adhesion layer (0.3 Å/s), and 50 ± 2 nm of Au (0.5 Å/s). Finally, lift-off was performed by soaking in acetone at 50 °C for 1 – 3 hours, followed by rinsing in isopropanol and blow drying.

### Transmission and reflection measurements

White light illumination in combination with an integrating sphere was used to measure the total transmittance of the NW network and ITO samples. A supercontinuum laser (Fianium SC400–4) was attenuated using a glass wedge and a ND 2.0 filter, after which it is focused by a f = 200 mm lens onto the samples. The samples are mounted in front of an integrating sphere (LabSphere). The transmitted light is collected from the sphere using a multi-mode collection fiber and fed into a spectrometer (Acton SpectraPro 300i) equipped with a Si CCD (Roper Scientific 7344–0001, cooled to −45 °C). The measured spectral range was 400 – 964 nm. Each reported spectrum is an accumulation of 250 spectra, each with 100 ms accumulation time. The spectra were normalized to the transmission through the bare glass, right next to the NWs/ITO on the same sample. The total reflection measurements on the completed devices were performed with the same settings. Here, a silver mirror is used as a reference.

### Sheet resistance measurements

The sheet resistance is determined from four-point probe measurements. For the NW networks, two probes were positioned on each contact pad. For the ITO the standard procedure using four equally spaced probes (spacing 1 mm) was used. IV-curves were measured with micro-positioning probes connected to a source meter (Agilent B2902A) by applying current (−10 to 10 mA, in 101 steps) through the samples using the outer probes and measuring the induced voltage with the inner probes. The sheet resistance is found from a linear fit through the data.

### Solar cell fabrication

ITO-coated glass substrates (200 nm thick ITO, Naranjo substrates) were cleaned by sonicating in acetone and soap-water followed by rinsing with normal water and sonicating in 2-propanol for few minutes. Silver nanowire substrates were cleaned only by mild rinsing in isopropanol to remove dust particles and were blow-dried using nitrogen.

A commercial formulation of high conductivity poly(3,4-ethylenedioxythiophene): poly(styrenesulfonate) (PEDOT:PSS), PH1000 was mixed with 5 wt% dimethylsulfoxide (DMSO) to enhance its conductivity and 0.2 wt% fluorosurfactant Zonyl FS-300 to facilitate wetting. The mixture was sonicated for 30 mins. This PEDOT:PSS formulation was then spin coated on top of ITO-coated substrates and Ag-nanowire-coated substrate at 6000 rpm to obtain a 30 nm thick film.

P3HT (>98% head to tail, *M*_*n*_ = 54,000–75,000 g mol−1, Plextronics, purchased from Aldrich) and PCBM (>99%, Solenne BV) in 1:1 weight ratio were dissolved in ortho-dichlorobenzene (*o*-DCB, 99%, Sigma-Aldrich) at a concentration of 40 mg/mL. The polymer solution was stirred overnight at 70 °C and was then filtered using a 1 μm-pore filter. The polymer was spin coated inside a nitrogen filled glovebox at 700 rpm to obtain a 220–230 nm thick film. Substrates were subsequently annealed inside a glovebox at 110 °C for 30 mins.

To complete the devices, 1 nm LiF, 100 nm Al and 500 nm Ag were evaporated using a circular shaped shadow mask (diameter = 1.9 mm). The thickness of all layers was measured using Veeco Dektak 150 Surface Profiler.

### Solar cell characterization

Current vs. voltage curves (I−V) were measured under simulated solar light (100 mW cm^−2^) from a tungsten–halogen lamp filtered by a Schott GG385 UV filter and a Hoya LB120 daylight filter using a Keithley 2400 source meter. The voltage range was −2 to 2 V. The short-circuit current density (*J*_*SC*_) was determined from the EQE data by multiplying with the AM1.5G solar spectrum and integration^44^.

EQE measurements were performed in a homebuilt set-up, with the devices kept in a nitrogen filled box and illuminated through an aperture of 1 mm diameter. Mechanically modulated (Chopper, Stanford Research, SR 540) monochromatic (Monochromator, Oriel, Cornerstone 130) light from a 50 W tungsten halogen lamp (Osram 64610) was used as probe light. A calibrated Si photodiode was used for the reference spectrum. The response was recorded as the voltage using a current preamplifier (Stanford Research Systems, SR570) connected to lock-in amplifier (Stanford Research Systems, SR830).

### Angle-resolved EQE measurements

A supercontinuum laser (Fianium, SC400-4) and a home-built prism-based monochromator were used to obtain fiber-coupled (multimode) monochromatic illumination (1.5 – 5.5 nm bandwidth, 57 – 65 μW). After the outcoupler, a 50 – 50 beam splitter was used to direct half of the power to a calibrated power meter (Thorlabs PM300) and half through a f = 200 mm lens onto the sample. The sample is mounted in the same nitrogen-filled box onto an automated rotation stage (HUBER 420–10724), and the generated photocurrent is measured by a source meter (Keithley 2612). In the experiment, the wavelength was scanned from 650 – 850 nm in steps of 5 nm. For each wavelength, the angle of incidence was scanned from 0 – 45 degrees in steps of 0.5 degrees. The measured photocurrent values are the average over 10 measurement points.

## Additional Information

**How to cite this article**: van de Groep, J. *et al*. Large-area soft-imprinted nanowire networks as light trapping transparent conductors. *Sci. Rep*. **5**, 11414; doi: 10.1038/srep11414 (2015).

## Supplementary Material

Supplementary Information
