# Supplementary material for: Large-area soft-imprinted nanowire networks as light trapping transparent conductors
Source: Sci Rep. 2015 Jun 19;5:11414. doi: 10.1038/srep11414 (PMC5155569; doi:10.1038/srep11414)
Supplement: Supplementary Information [file srep11414-s1.pdf]

Large-area soft-imprinted nanowire networks as light trapping transparent conductors

Supplemental Information

*Jorik van de Groep[1], Dhritiman Gupta[2], Marc A. Verschuuren[3], Martijn. M. Wienk[2], Rene A.J. Janssen[2] & Albert Polman[1]**

[1] Center for Nanophotonics, FOM Institute AMOLF, Science Park 104, 1098 XG Amsterdam, The Netherlands

[2] Departments of Applied Physics and Chemical Engineering & Chemistry, Eindhoven University of Technology, 5600 MB, Eindhoven, The Netherlands

[3] Philips Research Laboratories, High-Tech Campus 4, 5656 AE Eindhoven, The Netherlands

KEYWORDS: transparent conducting electrode, nanowire, scattering, plasmonics

CORRESPONDING AUTHOR

*polman@amolf.nl

S1: SCIL fabrication procedure

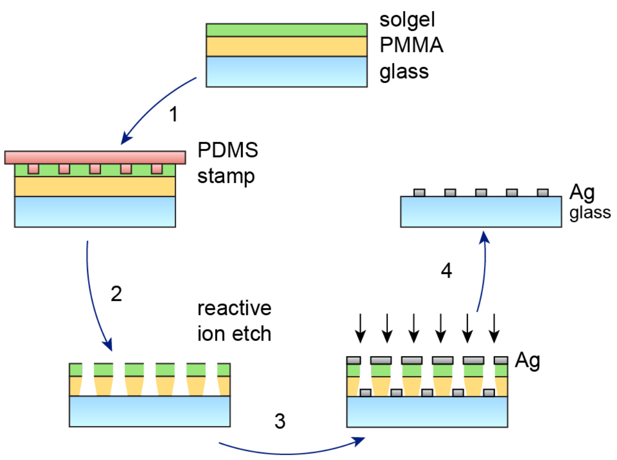

Figure S1 | Fabrication steps of substrate-conformal imprint lithography (SCIL) to form Ag nanowire networks.

SCIL is a high-resolution soft-imprint technique that uses a bi-layer PDMS stamp together with a silica-based sol-gel with minimized shrinking during the curing process, to obtain both high resolution and make it substrate conformal. Figure S1 shows the process steps of the SCIL fabrication of the NW networks. Low-molecular weight PMMA (yellow) is spin coated onto a glass substrate, followed by spin coating of ~65 nm of sol-gel (green). The PMMA will be used to perform lift-off. The PDMS stamp is applied without pressure, capillary forces pull the stamp into the sol-gel. After 30 minutes in ambient conditions, the sol-gel is cured and the stamp is removed. Next, reactive-ion etching (RIE) is used to etch through the residual layer of sol-gel and PMMA. Next, thermal evaporation was used to deposit 1 – 2 nm of Ge as a seed layer, followed by ~30 nm of Ag. Finally, the redundant metal was removed by dissolving the PMMA in acetone.

The timing of the RIE steps is critical and the optimum strongly depends on the filling fraction of the stamp. Over-etching causes the trenches in the sol-gel to widen due to the non-perfect anisotropy. Our stamp contains 40 patterns with different filling fraction. As a result, a large fraction of the patterns have been over-etched, resulting into wider wires and thus lower transmittance. The patterns can easily be optimized for transmission by reducing the REI etch times.

S2: Four-point probe measurements

To measure the sheet resistance of the networks, we assume that the resistance of the contact pads is very small compared to that of the NW network. To eliminate the effect of the contact resistance, a 4-point-probe experiment was performed (Fig. S2a). The outer 2 probes (red, channel 1) are used to apply a current through the network (-10 to 10 mA). The inner 2 probes (blue, channel 2) measure the induced voltage over the network.

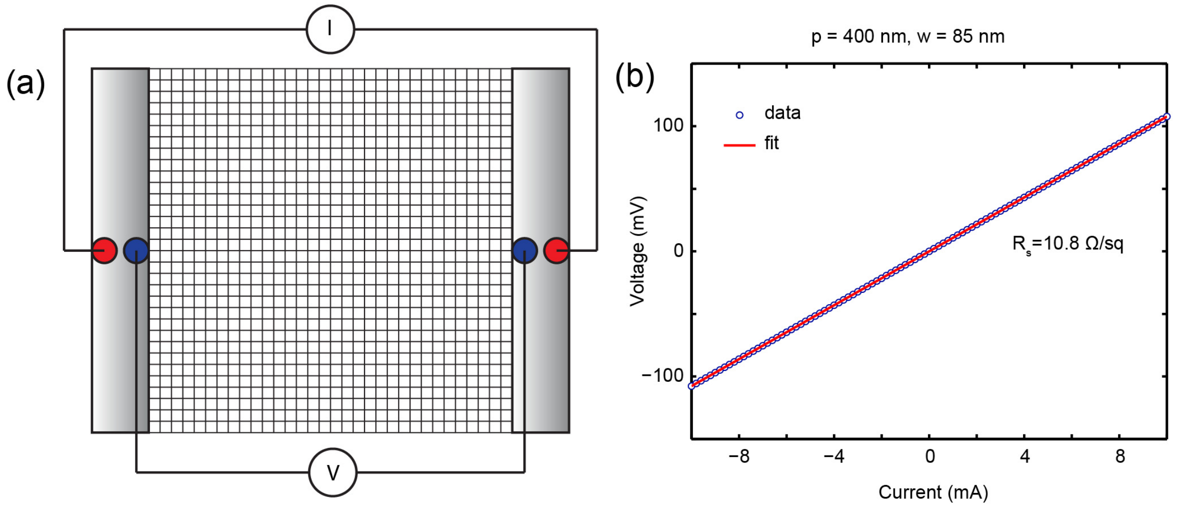

Figure S2 | Four-point probe measurement procedure. (a) Sketch of geometry for 4-point probe electrical measurements of the NW networks. (b) IV-curve for NW network (400 nm pitch) showing Ohmic behavior.

Figure S2b shows a typical IV curve, showing that the networks behave Ohmic (straight line) and the data (blue) can easily be fitted by a linear model (red). The slope gives the sheet resistance. For ITO, a standard four-point probe measurement is performed. For thin films, the sheet resistance can be obtained from the slope of the measured IV-curve multiplied by $\pi/\log(2)$. The data for ITO are the average of three measurements for each thickness.

S3: FDTD simulation details

Simulations for Fig. 1e,f were performed using periodic boundary conditions in the in-plane directions to mimic an infinitely large Ag NW network (30 – 130 nm width, 30 nm high, 500 nm pitch). For Fig. 1e, w = 65 nm was used. Perfectly Matched Layers (PMLs) were used in the vertical direction to simulate a semi-infinite glass substrate and air above the network. A broadband plane wave (400 – 965 nm) was launched from the top, and the transmittance into the glass substrate and the reflected power were monitored. Using the Lumerical far-field transformation routine, the power in each diffractive order was calculated. A 10 nm mesh, with a 1 nm refinement mesh around the NWs was used. Optical constants were taken from a Drude, Lorentz, and Debye model fitted to the data from Palik[1]. To convert the transmittance of the first interface (studied in Fig. 1e) into "normalized transmittance" (shown in Fig. 1f), the spectra are multiplied by the transmission coefficient of a glass – air interface for normal incidence and divided by the transmittance of a flat reference substrate.

Simulations of the completed device (w = 65 nm, h = 30 nm, p = 500 nm) were performed using rounded corners at the top of the NWs (radius of curvature 15 nm) to better match the experiment and improve the convergence. Thicknesses for the PEDOT:PSS and polymer layer were taken 30 and 230 nm respectively, similar to the case for the eigenmode calculations. The conformal coating of the PEDOT:PSS over the NW networks is also taken into account

(Fig. S4c), assuming a 30 nm thick layer on the side of the NWs and a 30 nm thick layer on top. The same optical constants as for the eigenmode calculations were used. A background index of 1.52 is used in order to take into account the presence of the glass superstrate. A 5 nm mesh, with a 1 nm refinement mesh around the NWs and PEDOT:PSS was used.

S4: Angle resolved EQE measurement details

Due to the dispersive nature of the prism in the home-built monochromator, the bandwidth increases for larger illumination wavelengths. Therefore, while scanning from 500 – 800 nm, the bandwidth is adjusted after scanning ~100 nm, in order to keep it roughly constant. To characterize the bandwidth of the monochromatic illumination in the angle-resolved EQE measurements, we measured the monochromator output at different wavelengths after each adjustment (Fig S3a). We find bandwidths in the range of 1.5 – 5.6 nm from a Lorentzian fit to the data.

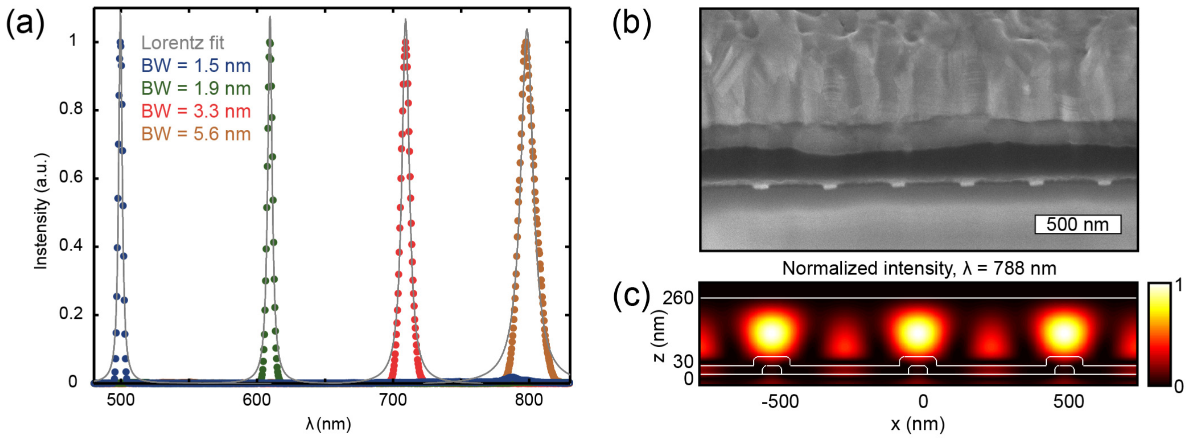

Figure S3 | Angle-resolved EQE details. (a) Measured monochromator output for several wavelengths across the scan range. Gray lines show Lorentzian fits through data. The bandwidth (BW) is listed as an inset, and is obtained from the FWHM of the fit. (b) FIB cross section showing the variations in polymer thickness across the device. (c) Simulated intensity profile, obtained from a y-z cross-cut perpendicular to the polarization (x-direction), through a

p = 500 nm device (w = 65 nm). The outline of the layer stack is shown in white. From top to bottom: glass, NWs, conformal coating of PEDOT:PSS, P3HT-PCBM, Al.

Focused Ion Beam (FIB) cross sections were performed in order to characterize the layer thicknesses of the devices as fabricated, as an input for the dispersion calculations (dashed lines, Fig. 3d,e). Large variations up to 65 nm in the thickness of the polymer can be observed (Fig. S3b).

Simulations of the field inside the complete devices (w = 65 nm, h = 30 nm, p = 500 nm) shows strong coupling to guided modes (Fig. S3c). The cross cut is taken perpendicular to the electric field polarization. Together with the mode profile (compare to Fig. 3b), this confirms that light couples to the TE_0 mode.

S5: Total reflection measurement of solar cells

Total reflection measurements on the completed devices were performed for comparison with the measured EQE (Fig. S4). Comparing the EQE of a NW network device (p = 500 nm) with that of the ITO reference device shows a large reduction (up to 0.2) in the visible (Fig. S4a). Embedding the NW networks in PEDOT:PSS instead of air makes the dielectric environment of the NW networks nearly uniform. This increases the backscattering of light, and thereby the reflectivity. Figure S4b shows that ~0.07 of the ~0.2 difference in EQE is a result of the increase in reflection. The remainder is due to enhanced parasitic absorption in the NWs, PEDOT:PSS and rear contact.

For wavelengths above the P3HT absorption edge ($\lambda > 650$ nm), the NW network device shows higher EQE (Fig. S4a) and lower reflection (Fig. S4b). The small peak in EQE

at $\lambda = 760$ nm roughly coincides with the spectral position of a sharp dip in reflection, which is a strong indication for mode coupling (spectral misalignment is a result of non-zero angle of incidence in the reflection measurements).

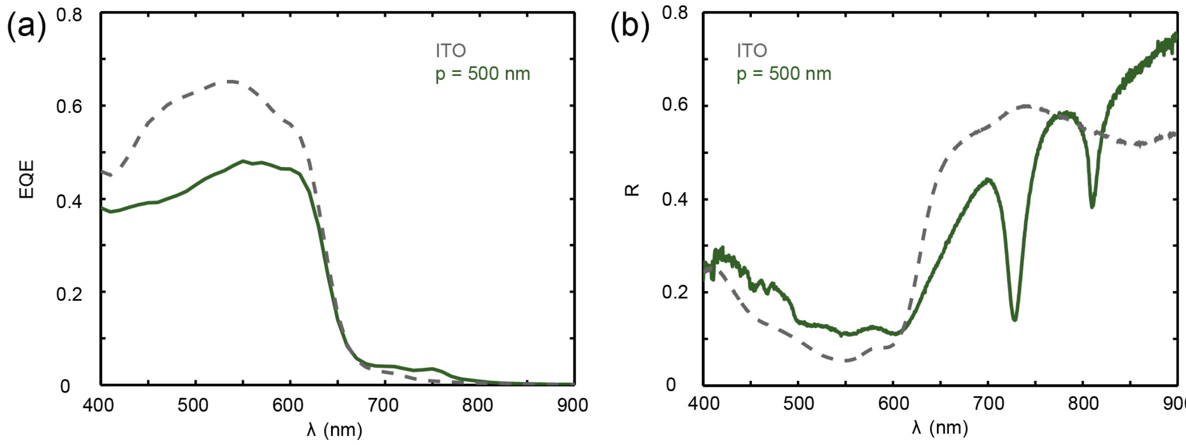

Figure S4 | Total reflection measurements. Measured EQE under normal incidence (a) and measured total reflection for $\theta = 6$ degrees (b) for the ITO reference device (gray) and a $p =$ 500 nm.

To further corroborate the role of mode coupling as a light trapping mechanism, Fig. S5 shows total reflection measurements for different pitch (Fig. S5a, w = 65 – 85 nm) and different wire widths (Fig. S5b, p = 600 nm). Varying the NW pitch alters the in-plane momentum obtained from diffractive coupling, giving rise to a spectral shift when coupling to a guided mode with a well-defined wavevector. Indeed, sharp dips at different spectral positions can be observed for different NW pitch (Fig. S5a). Note that the non-zero angle of incidence ($\theta = 6$ degrees) causes mode coupling to the same mode at different wavelengths, as apparent in Figs. 3e, S4b and S5a. Increasing the NW width increases the scattering cross section of the individual NWs, giving rise to an increase in backscattering in the visible as well as more efficient coupling to the guided mode in the NIR (Fig. S5b).

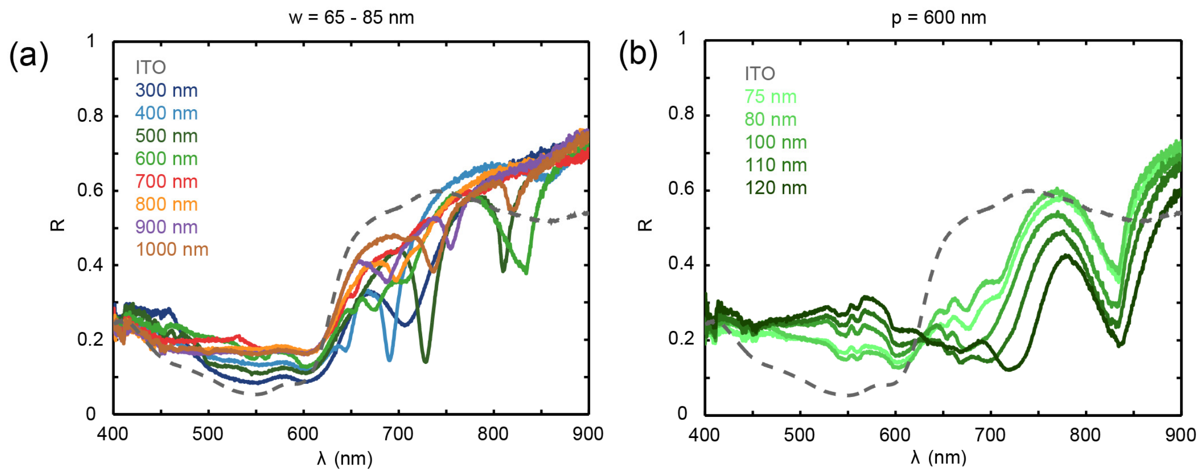

Figure S5 | Total reflection measurements for varying pitch and wire width. (a) Measured total reflection (θ = 6 degrees) for different pitched NW network devices (w = 65 – 85 nm). (b) Measured total reflection for different wire width (p = 600 nm).

S6: Influence of Ge seed layer on optical properties

The simulations in Fig. 1 neglect the presence of the 1 nm Ge seed layer, since this figure aims to describe the theoretical optimum performance that can be obtained with Ag NW networks (Fig. 1f). The inclusion of the Ge is an experimental necessity, which may be circumvented by further optimization of metal deposition methods. However, for low-aspect ratio structures such as those used in this work, the optical absorption of the Ge can be non-negligible. To demonstrate this, we simulate the optical absorption in the NWs with and without the 1 nm Ge layer. The results for a network with p = 500 nm, w = 65 nm, and h = 30 nm is shown in Fig. S6. Due to the strong absorption of Ge in the blue spectral range, the averaged absorption losses increase from 2.3% without to 5.4% with Ge seed layer (weighted for the AM1.5 photon density). This demonstrates that further optimization of the metal deposition techniques to relieve the necessity of the Ge seed layer is an important direction for future research.

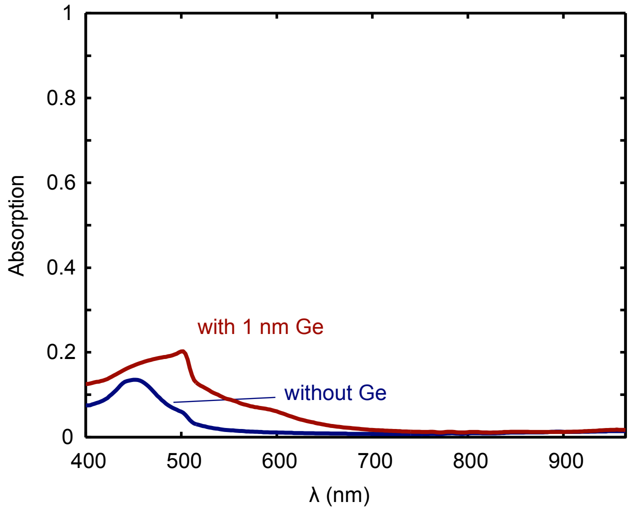

Figure S6 | Simulated absorption losses in nanowire networks with and without 1 nm Ge seed layer.

REFERENCES

1. Palik, E. D. *Handbook of Optical Constants of Solids*. Academic: New York, 1985.
